# Development of a Mediterranean Diet Recipe Index (MedRI)

**DOI:** 10.3390/nu17243868

**Published:** 2025-12-11

**Authors:** Sofia G. Florença, Filipa P. Costa, Raquel P. F. Guiné, Maria João Lima, Edite Teixeira-Lemos, Cristina A. Costa

**Affiliations:** CERNAS-IPV Research Centre, Polytechnic University of Viseu, 3504-510 Viseu, Portugal; sofiaflorenca@outlook.com (S.G.F.); filipa.pereira@esav.ipv.pt (F.P.C.); raquelguine@esav.ipv.pt (R.P.F.G.); amarocosta@esav.ipv.pt (C.A.C.)

**Keywords:** mediterranean diet, index, recipe evaluation, nutritional assessment, dietary adherence

## Abstract

**Background/Objectives:** The Mediterranean Diet (MD) is globally recognized for its nutritional, environmental, and cultural value. Although several indices assess adherence to the MD and its food environments, none evaluate the alignment of individual recipes with MD principles. This study aimed to develop and validate the Mediterranean Diet Recipe Index (MedRI), a novel scoring tool designed to quantify the concordance of recipes with MD guidelines. **Methods:** The MedRI was conceptualized through a comprehensive literature review and expert panel assessment, integrating two main dimensions: consumption context and recipe composition. The index evaluates ingredient selection, preparation methods, and food group inclusion, with criteria adapted to specific recipe categories. Validation was conducted using a structured questionnaire administered to 244 adults living in Portugal. Statistical analyses included descriptive statistics, Spearman correlations, intra-class correlation coefficients (ICCs), Cohen’s kappa, Chi-square tests, Cramer’s V, and principal component analysis (PCA). **Results:** Validation results demonstrated strong internal consistency and construct validity, confirming the reliability and applicability of the MedRI in assessing recipe alignment with MD principles. **Conclusions:** The MedRI thus represents a reliable and innovative tool to assess and promote culinary practices consistent with the MD. It holds potential applications in nutrition education, public health policymaking, and gastronomic research, supporting the advancement of sustainable and health-promoting dietary models.

## 1. Introduction

Food provides humans with energy, as well as macro- and micronutrients that support the growth, cellular processes, and metabolic and physiological functions of the body, thus making adequate nutrition fundamental for health and longevity [1,2].

However, in today’s world, non-communicable diseases (NCDs), such as type 2 diabetes, cardiovascular diseases, chronic respiratory diseases, cancer, obesity, and neurocognitive disorders, remain the leading causes of mortality and years of life lost. While genetic, metabolic, and environmental factors increase the risk of NCDs, behavioral determinants such as smoking, excessive use of alcohol, sedentary lifestyle, and unhealthy diets are key modifiable drivers of NCDs [1,2,3,4,5]. Food choices, in turn, are shaped by a complex interaction of numerous factors, such as environmental, biological, psychological, social, and cultural ones [6,7].

Dietary patterns are among the most important determinants of health, influencing both physical and mental well-being [2,8]. A healthy diet can be described as one that provides the necessary nutrients in adequate proportions, avoiding excesses and deficiencies, and contributing to disease prevention and overall well-being [1,9]. Dietary patterns are defined as “the quantities, proportions, variety or combinations of different foods and beverages in diets, and the frequency with which they are habitually consumed”, and can be represented as general descriptions, a priori diet scores, food guides, a posteriori formulations, or food and nutrient contents [10,11,12,13,14]. Consequently, nutrition research has evolved from nutrient-based analyses to pattern-based approaches, emphasizing food synergies and their cumulative health effects [11,13,15].

A variety of scoring systems and indices have been developed to assess dietary quality and adherence to healthy eating models. These can be classified into six categories: (1) food/nutrient quantity scores, (2) dietary diversity scores, (3) guideline-based scores, (4) nutrient quality scores, (5) health-based scores, and (6) adherence to a specific dietary pattern [16,17,18]. Such tools quantify diet quality either by rewarding the consumption of nutrient-dense or health-promoting foods, penalizing unhealthy or ultra-processed food intake, or measuring compliance with established dietary guidelines [11,14,18,19]. Recent advances also integrate environmental and sustainability criteria into dietary assessment, reflecting growing awareness of the link between human and planetary health [10,17].

Among healthy dietary models, the Mediterranean Diet (MD) stands out for its robust evidence base linking it to improved health outcomes and environmental sustainability. Originating from the regions surrounding the Mediterranean Sea, the MD reflects a historical convergence of geography, culture, agriculture, and social practices, traditionally centered on the “bread–olive oil–wine” triad. Over time, the MD has also experienced some changes, incorporating new foods and recipes until reaching the dietary pattern that is promoted nowadays as healthy, sustainable, and beneficial to human health [1,20,21,22].

The MD has been shown to offer many health benefits, including lowering the risk of all-cause mortality and preventing NCDs such as type 2 diabetes, cancer, heart diseases, neurological and cerebrovascular conditions, obesity, metabolic syndrome, and hypertension. Recent long-term cohort data further reinforce these benefits: a large 2024 study reported that higher adherence to the Mediterranean Diet was associated with significantly reduced all-cause mortality over more than two decades of follow-up [23]. Additionally, it positively influences blood sugar control, cognitive function, depression, liver diseases, and allergic conditions, and is linked to a longer life expectancy [1,2,4,15,20,21,22]. This dietary pattern is marked by a high intake and frequent consumption of fruits, vegetables, legumes/pulses, nuts, and unrefined, whole-grain cereals. The guidelines also recommend moderate consumption of fish, shellfish, eggs, white meat, and dairy products—preferably low-fat yogurt and cheese—while foods high in sugar and fat, as well as red and processed meats, should be eaten in small amounts. Furthermore, in the MD, the primary source of dietary fat is olive oil, and water is the main source of hydration. Wine is also suggested during meals but should be consumed in moderation [1,4,14,15,17,20,21,22,23].

The MD is not only a dietary pattern, but also includes a set of social, economic, environmental, and cultural elements. It is characterized by the use of local, traditional, seasonal, and minimally processed products, the protection of natural resources and biodiversity, as well as conviviality at mealtimes, sustainability, frugality, involvement in culinary practices, physical activity, and rest [4,18,24,25].

As referenced previously, cooking and culinary engagement is one of the important components of the MD. Several cooking methods and food combinations featured in the MD, for example, stews, casseroles, soups, and sautés with onion, olive oil, and tomato, also contribute to a higher bioavailability of health-promoted components [4,25]. In addition, the involvement in culinary practices promotes healthier dietary behaviors, as was shown in a study conducted by Mengi Çelik et al. [26]. Food preparation and cooking skills have been associated with a better diet quality, a higher intake of healthy foods, and a healthier state, since people who cook at home generally prepare food that is lower in fat, salt, sugar, and total energy than do those who eat out. Not only does cooking improve health, but it also allows the preservation of traditions and cultural identities, just as much as the act of eating together encourages connections, conversations, and sharing [27,28,29]. Along with cooking, recipes are a central aspect of cuisine as they are a tool for instructing how to prepare a dish as much as a way to preserve culture and gastronomy and promote knowledge sharing [30].

The world is constantly changing, which leads to transformations in society, economy, culture, and, consequently, in the food system. Globalization has encouraged the sharing of new foods, different cuisines, novel culinary techniques, and the modification of food habits, choices, and behaviors [31]. With so many changes taking place, several dietary patterns have also suffered erosion and a reduction in adherence, as is the case with the MD. The shift in eating habits, the greater consumption of unhealthy food products, the rise in sedentary lifestyles, and the increase in eating out and high consumption of fast food have led to an increase in all-cause mortality, NCDs, and obesity [32,33,34]. Therefore, it is important to value and encourage healthy and sustainable eating patterns, such as the MD, that provide health, social, economic, cultural, and environmental benefits [35].

In the scientific literature, there are numerous scores to assess people’s adherence to the MD [18,36,37], or to assess the food environment and its compliance with the MD [38,39]. However, to the authors’ knowledge, there is no index that assesses the alignment of a recipe with the MD. Therefore, this work aimed to design, develop, and validate a Mediterranean Diet Recipe Index (MedRI), a novel, multidimensional instrument to assess the alignment of recipes with the principles of the MD. By quantifying both the compositional and contextual dimensions of cooking, the MedRI provides an innovative means to evaluate, promote, and preserve Mediterranean dietary and cultural heritage, by evaluating the level of concordance of a food recipe with the MD, and subsequently to encourage adherence to the MD and home cooking.

## 2. Materials and Methods

### 2.1. Development of the Mediterranean Diet Recipe Index (MedRI)

The MedRI (Table 1) was developed based on a comprehensive literature review conducted on three main themes: (1) the core principles and guidelines of the MD, (2) existing scores and indices to assess adherence to the MD, and (3) scores and indices to assess food environments and their compliance with the MD.

The expert panel included eight specialists chosen for their proven experience in Mediterranean Diet research, nutrition science, food technology, gastronomy, and sustainability. Selection was based on academic credentials, relevant peer-reviewed publications, and institutional affiliation. Consensus among the experts was reached through multiple reviews, scoring of components, and structured discussions until a majority agreement was achieved. The MedRI is an analytical instrument designed to quantify an individual’s level of adherence to the MD and the degree of adequacy and suitability of a recipe with the MD standards. This index assigns a composite score based on two dimensions: I. Recipe composition and II. Consumption context. The first part integrates elements of the recipe, namely, preparation methods and food groups. The second part captures the social and environmental aspects of the meal preparation and consumption, including the selection criteria for the ingredients used in the recipe. Each item was assigned a point value based on its conformity with MD principles, as detailed in Table 1. The rationale for including each component of Table 1 is based on both nutritional and cultural aspects of the Mediterranean Diet. Components that are central and characteristic of the MD—such as vegetables, legumes, fruits, whole grains, olive oil, fish and seafood, aromatic herbs, and traditional preparations (soups, stews, sofrito)—were included because they represent core dietary patterns and historically established culinary practices of Mediterranean cuisine. Conversely, foods shown to be inconsistent with MD principles (processed meats, ultra-processed products, red meats in high amounts, and added sugar) were included as negative markers to help identify dietary deviations from MD patterns.

Similarly, the point values were determined by how well they aligned with MD principles: components fundamental to the MD received +2 points, those acceptable in moderation received +1 point, and foods or sourcing practices incompatible with MD guidelines were assigned −1 point. Butter and margarine were assigned +1 point to reflect their occasional but limited use in certain Mediterranean culinary traditions, acknowledging that although they are not primary fats in the Mediterranean Diet (where olive oil is preferred), they may be included in moderation without significantly reducing MD adherence. These values were not arbitrary; they were refined through expert consensus and empirically calibrated against a benchmark of 100 traditional Mediterranean recipes to ensure an accurate representation of authentic MD culinary patterns. The index considers the category of the recipe—breakfast, soup, main dish, vegetarian dish, vegan dish, salad, sauces, side dish, and dessert—with the different components of the MedRI being assigned to each category when deemed appropriate. Prior to the statistical validation, the MedRI component identification and the categorization process were conducted and reviewed by a multidisciplinary panel of food science experts.

To set classification thresholds, scoring cut-offs for the red, yellow, and green categories were empirically determined using a curated reference set of 100 traditional Mediterranean recipes. This calibration sample helped us establish realistic boundaries that reflect true Mediterranean culinary patterns rather than arbitrary numbers. To do this, we compiled a database of 100 traditional Mediterranean recipes from validated sources, including MD recipe books [40,41,42] and the Portuguese official MD website (http://www.dietamediterranica.pt (accessed on 20 October 2025)) [43]. For each recipe category, we analyzed the score distribution from these reference recipes to identify natural scoring ranges. We calculated the minimum, maximum, mean, median, and quartiles, and used these statistical measures to define the “red,” “yellow,” and “green” classification ranges.

In regard to the scoring system, the MedRI assigns a dual score, the individual adherence score to an MD (Table 2), which includes the points obtained from parts I and II, reflecting how closely a participant’s cooking and ingredient choices align with MD practices, and the recipes’ adequacy score to an MD (Table 3), which only comprises the points assigned by part I, and evaluates the intrinsic conformity of the dish with MD standards. Both scores are classified into three levels—red (low adherence/adequacy), yellow (moderate adherence/adequacy), and green (high adherence/adequacy)—based on the total points earned, adjusted for each recipe category.

### 2.2. Instrument of Data Collection

The validation of the MedRI was carried out using a questionnaire, approved by the Ethical Committee at the Polytechnic University of Viseu, with reference N.º 06/SUB/2025. The questionnaire included questions to validate the index, as well as additional questions to characterize the study population. The final questionnaire contained three sections, with a total of 18 questions. The first section consisted of the sociodemographic data: (1) Age, (2) Gender, (3) Level of education, (4) Living environment, (5) District, (6) Weight, (7) Height, and (8) How often do you usually exercise? The second part collected information regarding recipes and the context in which they were made, with questions such as (9) Indicate a recipe that you have prepared recently (from your daily life, within your family, alone or in other contexts), (10) In what context was the recipe consumed? (11) How often do you make this recipe? (12) Do you consider this recipe to be part of the Mediterranean diet? (13) Regarding the positive or negative impact of this recipe, indicate which parameters you consider relevant, (14) Ingredients (Please indicate the ingredients used and their quantities in grams or liters, as appropriate), and (15) Preparation method (Please indicate all the steps for making the recipe, numbering them). The third and final section was regarding the ingredients utilized in the recipe consisting of questions about selection criteria and accessibility, namely, (16) Of the ingredients used in the preparation of the recipe, what are your criteria for choosing them? (17) If any of the ingredients mentioned are difficult to obtain or purchase, please indicate which ingredient(s) are more difficult to obtain, and (18) What are the reasons why ingredients are difficult to obtain? This structure allowed simultaneous assessment of the quantitative scoring of recipes and the qualitative rationale behind participants’ ingredient choices and cooking behaviors.

### 2.3. Data Collection

The data collection occurred between March and August of 2025, with the abovementioned questionnaire being presented to willing participants who were adults (18 years or older) residing in Portugal and in a convenience sample. The survey was carried out online using the Google Forms software (Google, Mountain View, CA, USA), and the dissemination occurred through social media and email platforms. No financial or material compensation was offered for participaion; all participation was entirely voluntary. Furthermore, participants were assured of the anonymity and confidentiality of the data and were only able to access the questionnaire after accepting the study information, namely, objective, methodology, data use, storage, and destruction, and giving informed consent.

To evaluate the internal reliability of the MedRI, a minimum sample size of 100 respondents was required, following recommendations for reliability and instrument validation analyses using ICC and agreement statistics [44,45]. In total, 244 people completed the questionnaire, meeting the required sample size.

### 2.4. Data Analysis

Data statistical analysis was conducted using SPSS software version 27 (IBM, Inc., Armonk, New York, NY, USA) and Excel 365 (Microsoft, Redmond, WA, USA). In addition to performing statistical tests, we applied each method with a specific validation purpose; namely, ICC to assess the reliability of MedRI components, Cohen’s kappa to examine agreement between categorical classifications, Chi-square and Cramer’s V to test associations across sociodemographic and recipe variables, and PCA to evaluate construct validity and explore latent patterns in ingredient selection. These rationales are subsequently explained in detail in the following paragraphs. Because the data consisted predominantly of categorical and ordinal scoring outputs rather than continuous variables, normality testing was not required, and non-parametric and agreement-based methods were applied.

Prior to the data analysis, two new variables were created from existing data: Age group, which consisted in grouping the variable Age into four classes: adults (18–30 years), middle-aged adults (31–50 years), senior adults (51–65 years), and older adults (aged 66 years or more); and BMI, which was calculated as weight divided by height squared (kg/m^2^) and classified according to the World Health Organization criteria—underweight (<18.5 kg/m^2^), normal weight (18.5–24.9 kg/m^2^), overweight (25.0–29.9 kg/m^2^), and obesity (≥30.0 kg/m^2^).

For the data analysis, cross-tabulation and descriptive statistics, such as frequencies, minimum values, maximum values, mean values, and standard deviations, were calculated.

To measure and evaluate the relationships between variables, the Spearman correlation coefficient was used. A value close to 1 indicates a strong positive correlation, while a value near −1 indicates a strong negative correlation. Absolute values of the correlation coefficient less than 0.2 suggest a weak correlation, values from 0.20 to 0.49 suggest an acceptable correlation, and values greater than 0.50 indicate a good correlation [46,47].

For the MedRI validation assessment, several statistical analyses were conducted. The MedRI’ reliability was tested using the Intra-class Correlation Coefficient (ICC) with a two-way mixed model, which was selected to measure the consistency of scoring across different raters and recipe instances. ICC evaluates the degree to which repeated measurements of the same construct yield consistent results, with higher values indicating stronger agreement. ICC values < 0.50 indicate poor agreement, values between 0.50 and 0.75 moderate agreement, between 0.75 and 0.90 good agreement, and values greater than 0.90 represent an excellent reliability [48].

The agreement between the classification of the recipes’ adequacy score and the individual adherence score to an MD, based on the appropriate cut-off points available in Table 2 and Table 3, was assessed with Cohen’s kappa. This coefficient was selected because it evaluates the degree of agreement between categorical classifications while accounting for the level of concordance expected by chance, providing a robust measure of classification reliability. Cohen’s kappa values ≤ 0 show that there is no agreement, values between 0.01 and 0.20 show a slight agreement, between 0.21 and 0.40 show a fair agreement, between 0.41 and 0.60 show a moderate agreement, between 0.61 and 0.80 show a substantial agreement, between 0.81 and 0.99 show an almost perfect agreement, and a value of 1 indicates a perfect agreement [48,49].

Additionally, cross-tabulation analysis, Chi-square test, contingency coefficient (CC), and Cramer’s V were used to measure the strength and significance of the associations between the categorical variables. These tests were selected to determine whether relationships existed between recipe attributes, sociodemographic variables, and MedRI scoring categories, allowing us to evaluate whether adherence patterns were systematically associated with specific participant characteristics. The Chi-square test evaluates if there is a significant statistical association between two variables; thus, a ρ-value greater than 0.05 indicates that the association is not statistically significant. The CC is based on the Chi-square test and varies between 0 and 1, where 0 indicates independence, and 1 a strong association, and values between 0.1 and 0.19 show a weak association, between 0.2 and 0.29 a medium association, and above 0.3 a strong association [50]. The Cramer’s V coefficient varies from 0 to 1; for V ≈ 0.1, the association is considered weak; for V ≈ 0.3, the association is moderate; and for V ≈ 0.5 or over, the association is strong [51] between recipe attributes, sociodemographic variables, and MedRI scoring categories, allowing us to evaluate whether adherence patterns were systematically associated with specific participant characteristics. Lastly, a principal component analysis (PCA) was applied to the variables related to ingredient selection criteria to assess construct validity by identifying latent structures underlying decision-making patterns. PCA was selected because it reduces dimensionality and reveals natural clustering in the data, allowing us to verify whether ingredient choice behaviors are grouped in a manner consistent with Mediterranean Diet principles. To further examine the factor structure, an exploratory analysis using Varimax rotation was performed, employing an absolute factor loading cut-off of >0.40 to suppress small coefficients. Data suitability was confirmed through Bartlett’s Test of Sphericity (ρ < 0.05) and the Kaiser–Meyer–Olkin (KMO) Measure of Sampling Adequacy, with KMO values below 0.50 considered unacceptable, 0.50–0.69 acceptable, 0.70–0.79 good, 0.80–0.89 very good, and ≥0.90 excellent. Factors with an eigenvalue of ≥1.0 were retained, and the total explained variance was recorded [52,53].

The level of significance considered in all the analyses conducted was 95%.

## 3. Results

### 3.1. Sociodemographic Data

The participants’ sociodemographic characteristics are described in Table 4 and Table 5. The final sample comprised 244 adults residing in Portugal, with an average age of 44.99 ± 13.56 years, a minimum age of 18, and a maximum age of 74 years (Table 4). The average height and weight of the respondents were, respectively, 165.79 ± 7.97 cm and 69.23 ± 14.90 kg (Table 4). The minimum height was 147.00 cm and the maximum height was 188.00 cm. Regarding weight, the minimum value reported was 38 kg and the maximum value was 168 Kg. The BMI of the participants ranged from 15.61 kg/m^2^ to 59.52 kg/m^2^, with a mean value of 25.11 ± 4.82 kg/m^2^ (Table 4).

Most participants were middle-aged adults (43.4%), female (79.1%), held a university degree (48.0%), and lived in urban areas (54.1%) (Table 5). The majority of the study population had a normal weight according to BMI (53.3%), but a high percentage were overweight (28.3%) or obese (12.7%) (Table 5). Regarding physical activity, most respondents exercise moderately up to two to three times a week (37.3%), while 6.6% never exercise and 16.8% do so sporadically, less than once a week.

More than half of the participants are from urban areas, mainly living in Viseu (*n* = 93), Lisbon (*n* = 31), Porto (*n* = 25), and Faro (*n* = 21).

**Figure 1:**
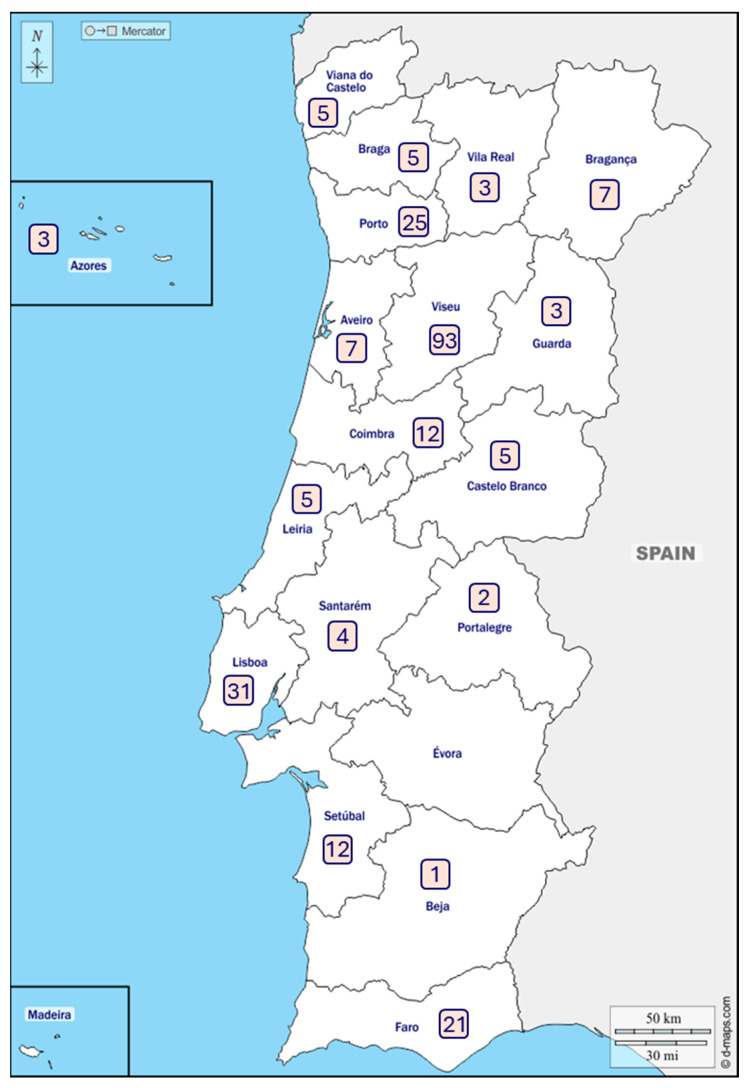
Number of participants per district.

Évora and the Autonomous Region of Madeira were the only districts without representation, ensuring broad territorial coverage across mainland Portugal (Figure 1). The central region had a higher level of representation than other areas of Portugal.

### 3.2. Reliability and Validity Analyses

The statistical validation of the MedRI was achieved by using ICC analysis, the Spearman correlation coefficient, Cohen’s kappa, the Chi-square test, the contingency coefficient, and Cramer’s V.

The ICC analysis has shown a moderate reliability for the individual adherence score to an MD (ICC = 0.659, ρ < 0.01) and an excellent reliability for the recipes’ adequacy score to an MD (ICC = 0.924, ρ < 0.01). These results confirm that the MedRI yields consistent and replicable evaluations when applied to different datasets or raters, particularly regarding the intrinsic composition of recipes.

Cohen’s kappa was used to measure the agreement between the classification of the recipes’ adequacy score and the individual adherence score to an MD (Table 6). Additionally, cross-tabulation analysis, CC, and Cramer’s V were used to analyze the strength of the associations (Table 6).

In regard to the Chi-square test, the association between scores is only significant for the categories main dish (ρ < 0.001) and dessert (ρ = 0.028). The CC shows a strong relationship for the associations derived from the Chi-square test (main dish CC = 0.640; dessert CC = 0.612) (Table 6).

The Cramer’s V coefficient shows that the associations for main dish (V = 0.590), vegetarian dish (V = 0.595), vegan dish (V = 0.577), and dessert (V = 0.775), all have a coefficient above 0.5, which corresponds to a strong relation between both scores (Table 6).

The Cohen’s kappa statistical measure indicates that, between the categorized classification of the recipes’ adequacy score and the individual adherence score to an MD, there is a slight agreement for main dish (κ = 0.059), vegetarian dish (κ = 0.006), vegan dish (κ = 0.200) and salad, sauces, and side dish (κ = 0.010), and a fair agreement for desserts (κ = 0.273) (Table 6).

Overall, these analyses confirm that the MedRI demonstrates good internal reliability and construct validity, with the strongest consistency observed in recipe-based classifications. The partial divergence between the individual and recipe dimensions reflects the conceptual design of the tool—capturing not only nutritional composition but also behavioral and contextual aspects of Mediterranean culinary practices.

### 3.3. MedRI Scoring and Questionnaire Data

The MedRI was applied to the data collected through a questionnaire, resulting in two scores: the individual adherence score to an MD, consisting of the consumption context dimension and the recipe composition dimension, and the recipes’ adequacy score to an MD, encompassing the recipe dimension.

The data from the questionnaire shows that the majority of the respondents submitted recipes regarding main dishes (*n* = 163), whereas vegan dishes (*n* = 4), breakfast (*n* = 8), and desserts (*n* = 8) were the least frequent categories of recipes included in the responses (Table 7). This predominance of main dishes reflects the central role of these preparations in Mediterranean daily meals and their cultural importance within the Portuguese context.

A high proportion of participants scored a yellow or green classification in the MedRI individual adherence to an MD, thus indicating a moderate to high individual adherence to an MD.

In terms of individual adherence to the Mediterranean Diet, most respondents achieved moderate to high adherence levels, corresponding to the yellow and green categories of the MedRI (Table 7). Only a small fraction of participants (8.6%) scored within the red (low adherence) category, exclusively in the main dish group. For breakfast and soup, all participants achieved a green classification, indicating full alignment with MD principles in these categories, likely due to their traditional simplicity and the high prevalence of characteristic ingredients (e.g., olive oil, vegetables, whole grains).

The results from Table 8 highlight that, for the breakfast, soup, and salad, sauces, and side dish categories, the recipes submitted by the participants had a MedRI classification of yellow and green, which indicates a moderate to high adequacy of the recipes to an MD. Furthermore, the soup category had, of all the recipe categories, the highest recipe adequacy to an MD, with a value of 76.5% (Table 8).

The recipes included in the questionnaire covering main dishes, vegetarian dishes, vegan dishes, and desserts were classified according to their adequacy to the MD, ranging from green—high adequacy—to red—low adequacy. For these four categories, the highest percentage of recipes fell into the yellow classification, indicating that the majority of the recipes from these categories had a moderate adequacy to the MD (Table 8).

Neither the vegan dish nor the dessert category included any recipe in the green classification, with recipes being exclusively in the yellow and red classes, thus indicating a moderate to low adequacy to an MD (Table 8).

In Table 9, the correlation between sociodemographic data and MedRI scores is represented. The results show that the variable age class is positively and significantly correlated (ρ < 0.05) with the MedRI individual adherence score to an MD (ρ = 0.130). The gender has shown a negative and significant correlation (ρ < 0.05) with both MedRI scores, the individual adherence score to an MD (ρ = −0.162) and the recipes’ adequacy score to an MD (ρ = −0.150). In regard to the education level, the data indicates a positive and significant correlation at the 0.01 level, for both MedRI scores, the individual adherence score to an MD (ρ = 0.239) and the recipes’ adequacy score to an MD (ρ = 0.221) (Table 9). No significant correlation was found for the remaining sociodemographic variables—living environment, district, BMI class, and exercise. All the correlations, except for education level, are considered poor associations, with values < 0.2. The correlations between education level and the MedRI scores are acceptable associations since the values are between 0.20 and 0.49 (Table 9).

The relationship between the consumption context and MedRI scores (Table 10) further supports the internal validity of the tool.

The data indicates that the meal sharing dimension is positively and significantly (ρ < 0.01) correlated with choosing local products (ρ = 0.227) and the MedRI individual adherence score (ρ = 0.209) and negatively (ρ < 0.05) correlated with certified products (ρ = −0.135). The positive correlations are considered acceptable, whereas the negative correlation indicates a poor association (Table 10).

The selection of seasonal ingredients has a significant (ρ < 0.05) correlation with fresh (ρ = 0.326) and certified products (ρ = 0.136), as well as with the MedRI individual adherence score (ρ = 0.475) and recipes’ adequacy score (ρ = 0.161) (Table 10).

The selection of fresh products has also been positively correlated with both the MedRI individual adherence score (ρ = 0.478) and recipes’ adequacy score (ρ = 0.194) (Table 10).

The MedRI individual adherence score has shown a positive and good correlation with the MedRI recipes’ adequacy score (ρ = 0.869). The value of this correlation indicates a good association (Table 10). This strong correlation indicates that, although conceptually distinct, the contextual and compositional dimensions are mutually reinforcing—validating the index’s integrated approach.

### 3.4. Factor Analysis

An exploratory factor analysis using the PCA method with Varimax rotation was conducted to explore the latent structure of the “ingredient selection criteria” variables included in the MedRI and to assess its construct validity. The adequacy of the data for factor analysis was verified through the KMO Measure of Sampling Adequacy and Bartlett’s Test of Sphericity statistical tests. The results showed a KMO of 0.532, which indicates an acceptable data suitability, and a significant Bartlett’s Test of Sphericity (ρ < 0.001), confirming that the dataset is suitable for factor analysis. Based on these results, a factor analysis was conducted, resulting in six factors, which, in total, explained 61.19% of the variance. Each factor was interpreted based on the variables with loadings above 0.40 after Varimax rotation, which enhances interpretability by minimizing cross-loadings. Factor 1 consists of three criteria—national, seasonal, and fresh products—which explained 15.16% of the total variance and presented an eigenvalue of 1.971 (Table 11). Factor 2 is represented by two criteria—Protected Designation of Origin (PDO) and Protected Geographical Indication (PGI)—which explained 10.44% of the total variance and presented an eigenvalue of 1.358 (Table 11). Factor 3 is constituted by three criteria—organic products, direct from the producer, and ultra-processed foods—explaining 10.03% of the total variance and presenting an eigenvalue of 1.303 (Table 11). Factor 4 consists of two criteria—foreign and own production products—which explain 8.94% of the total variance and present an eigenvalue of 1.162 (Table 11). Factor 5 is represented by two criteria—economic and non-certified products—which explain 8.49% of the total variance and present an eigenvalue of 1.103 (Table 11). Factor 6 consisted of one criterion—buying in local markets—which explains 8.14% of the total variance and presents an eigenvalue of 1.058 (Table 11).

Overall, these six factors collectively describe the multidimensional nature of ingredient selection within Mediterranean cooking practices, encompassing not only nutritional and sensory attributes but also socioeconomic, cultural, and environmental considerations. The extracted structure supports the conceptual framework of the MedRI, in which ingredient choice is influenced by interrelated drivers such as freshness, locality, tradition, affordability, and sustainability—all of which are consistent with Mediterranean Diet principles.

## 4. Discussion

The traditional MD was shaped over centuries by the interaction of geographic, cultural, social, and economic factors among populations living around the Mediterranean basin. This interplay of environment, tradition, and necessity produced a dietary model centered on plant-based foods, local production, and conviviality, which UNESCO recognized in 2013 as an Intangible Cultural Heritage of Humanity [54]. The MD thus represents not only a nutritional paradigm but also a cultural and ecological model that promotes health, biodiversity, and sustainability.

In the countries where MD is a traditional dietary pattern, numerous historical and modern recipe collections reflect the evolution of Mediterranean gastronomy and its health-promoting principles [55]. However, it is important to investigate to what extent the recipes that people prepare in their home environment for consumption in a day-to-day and/or friend/family enlarged context are compatible with the principles of the MD. The scores validated to measure adherence to MD are based on the intake of certain foods (e.g., food frequency questionnaires); for example, the PREDIMED score, composed of 14 items [56,57,58,59]. Boronat et al. [60] compared ingredients in culinary preparations across some Mediterranean countries (Egypt, Lebanon, Italy, Portugal, and Spain), revealing five key ingredients shared across countries: olive oil, garlic, onion, tomato, parsley, and black pepper [60]. Yet to date, there is no validated tool to systematically evaluate the degree to which recipes adhere to the MD. To our knowledge, the MedRI is the first validated index specifically created to assess Mediterranean Diet adherence at the individual recipe level, thus closing the methodological gap between food-frequency dietary adherence indices and tools evaluating Mediterranean food environments. The present study addressed this gap through the development and validation of the Mediterranean Diet Recipe Index (MedRI), based on a multidimensional approach that aligns with the holistic nature of the MD, where food quality, social interaction, and sustainability coexist as inseparable pillars of well-being. The MedRI therefore integrates two fundamental dimensions of the MD: one focused on the ingredients and preparation methods, and another on the consumption context dimension, capturing social, cultural, and environmental aspects of meal preparation and sharing [54].

From a nutritional perspective, the MedRI’s scoring criteria reflect the extensive scientific evidence linking the core components of the MD to health outcomes [61]. Some basic principles of this dietary pattern include prioritizing the consumption of fruits, vegetables, whole grains, legumes, nuts, seeds, and healthy fats like extra virgin olive oil. Regarding alcoholic beverages, moderate consumption is recommended, and when present, red wine is preferred. Conversely, ultra-processed foods, refined grains, and foods with added sugars should be avoided or eaten with strict moderation. For example, poultry, eggs, dairy products, fish, and seafood should be consumed several times per week, whereas red meat and sweets should be limited to no more than once per week [54,55,62]. These principles were all considered when scoring the recipes, assigning positive or negative points based on their alignment with MD standards. Higher positive scores were given to soups, stews, casseroles, sofrito, vegetables, fresh fruits, legumes, whole grains, nuts, olive oil, aromatic herbs, eggs, some low-fat dairy products, white meat, fish, and seafood. Conversely, negative points were assigned to processed meats or sugary foods. The elements used for recipe classification have been validated through statistical analysis and can now serve as a reference for future studies.

The validation of the recipe scores based on food elements relies on scientific evidence that foods of the Mediterranean Diet (MD) contribute to improved health. Extra virgin olive oil has been thoroughly researched for its health benefits, as demonstrated in studies by Huang et al. [63] or Ussia et al. [64]. Fish and nuts contain important unsaturated fatty acids with proven positive health effects [65,66]. Dairy products, especially low-fat ones rich in calcium [67], and functional yogurt, which contains beneficial live microorganisms [68], have also proven to be valuable for promoting health. Whole grains include, among other macro- and micronutrients, dietary fiber, which has demonstrated significant benefits for the health of the gastrointestinal system and for reducing the risk of various chronic non-communicable diseases [69,70,71]. Vegetables and fruits are among the most recommended healthy foods because they contain multiple macro- (including dietary fiber) and micronutrients (vitamins, minerals), as well as bioactive molecules like phenolic compounds with important antioxidant properties [72,73,74,75,76].

The MD is based on principles that regulate portion and serving sizes for different food groups to ensure a balanced intake of beneficial nutrients. It includes a diverse range of foods containing various nutrients that work together to enhance health. Therefore, the benefits of the MD cannot be attributed to a single food or ingredient alone. Instead, it is the combination of nutrients, along with other external factors, that makes the MD healthy. In fact, regardless of the specific foods and ingredients, it has been suggested that the way people in Mediterranean areas prepare and consume meals also contributes to the positive effects of this dietary pattern. The MD dietary pyramid starts with a foundation that emphasizes social, cultural, and physical activity aspects. Following the MD involves lifestyle choices such as regular physical activity, adequate rest, and socialization, as well as considerations related to food production and sourcing—eating seasonal foods, preferring traditional, local options that support biodiversity and are environmentally friendly.

Finally, culinary activities should also be considered, with cooking methods such as boiling or stewing preferred over frying [55,77,78,79]. These specificities were also incorporated into the developed score, within the dimension of the consumption context, assigning positive points to aspects like sharing meals, buying ingredients from local markets or producers, using fresh and seasonal ingredients, or choosing certified products (organic farming, PDO—Protected Designation of Origin). Statistical analysis also validated this dimension of the score.

Considering the composition of the recipes and their allocation to the standard MD, different classes for assessing recipe adequacy were established based on the scores obtained. A similar procedure was also used to assess individuals’ adherence to the MD, taking into account two dimensions: recipe and consumption context. These were linked to sociodemographic variables, showing statistically significant relationships with education level, gender, and age. Regarding education, it has been observed that more educated individuals tend to engage more in healthier practices, especially those with higher levels of food literacy [80,81,82]. Gender disparities in adherence to the MD have been reported, with women generally showing higher adherence than men, possibly due to concerns about practicing a healthy diet or for aesthetic reasons [83,84,85]. Since recipe preparation is also included, this finding is not surprising. As for age, adherence to the MD has been evaluated across various age groups—from children to older adults—including adolescents, young adults, and adults. These studies report age-related differences, with the role of parents particularly highlighted among younger individuals [86,87,88,89,90].

This study using the MedRI on 244 Portuguese adults provided valuable insights into current dietary patterns and recipe compositions. Most participants had moderate to high adherence to Mediterranean Diet (MD) principles, especially by using fresh, seasonal, and locally sourced ingredients. However, the presence of ultra-processed foods and non-national products in some recipes highlights ongoing challenges and opportunities to improve the preservation of traditional dietary habits amidst globalization and modern food environments. Sociodemographic analysis further revealed that middle-aged, educated, urban women were the main respondents, most of whom had a normal BMI and engaged in moderate physical activity. These traits influence dietary choices and home-cooking habits, both of which are linked to greater adherence to the MD. These results align with previous research emphasizing the importance of education, food literacy, and cultural engagement in maintaining Mediterranean eating habits.

Despite the strength of the methodological and statistical procedures used, some limitations of this study must be recognized. Data were gathered through a convenience sampling method via online channels, which may have introduced selection bias. The final sample mainly consisted of middle-aged, female, highly educated participants—groups that tend to be more aware of healthy eating habits and more likely to cook at home. This demographic profile could therefore lead to an overestimation of Mediterranean Diet adherence and restrict the generalizability of the findings to the broader Portuguese population. Additionally, the sample had a disproportionate number of main dish recipes, with fewer breakfast, vegan, and dessert recipes, which might reduce the representativeness of the MedRI’s validation across all recipe types. Future validation will intentionally expand recruitment to include more heterogeneous socio-demographic groups (including varying ages, genders, education levels, and cultural backgrounds) and will incorporate cross-country testing in both Mediterranean and non-Mediterranean regions to improve external applicability, reduce potential inflation of MD adherence, and enhance the robustness of the MedRI for international use

Nevertheless, the development and validation of the MedRI represent a pioneering step in connecting culinary practice, nutrition science, and sustainability assessment. By measuring how recipes embody the core values of the Mediterranean Diet—nutritional balance, cultural heritage, and environmental responsibility—the MedRI offers a practical, evidence-based tool with strong potential for research, education, and public health policy. This innovative approach transforms traditional dietary assessment into a more dynamic, culturally relevant, and behaviorally focused evaluation of healthy eating patterns. This study also presents several strengths that reinforce its contribution. First, the MedRI is, to our knowledge, the first index to evaluate Mediterranean Diet adherence by integrating both recipe composition and consumption context within a single analytical framework. Second, the classification thresholds were empirically derived through calibration with 100 traditional Mediterranean recipes, ensuring cultural authenticity and methodological robustness. Third, the index demonstrated strong reliability through appropriate validation analyses, confirming its reproducibility and consistency. Finally, the MedRI offers a versatile and adaptable framework that may support future applications in nutritional research, culinary education, food innovation, and dietary assessment across both Mediterranean and non-Mediterranean settings.

## 5. Conclusions

This study successfully developed and validated the Mediterranean Diet Recipe Index (MedRI), a novel, comprehensive tool to assess the alignment between culinary recipes and the core principles of the Mediterranean Diet (MD). By integrating two complementary dimensions—recipe composition and consumption context—the MedRI provides a multidimensional evaluation framework that captures not only nutritional quality but also social, cultural, and environmental aspects inherent to the Mediterranean lifestyle.

The statistical validation confirmed the reliability, internal consistency, and construct validity of the MedRI, demonstrating its capacity to differentiate levels of recipe adequacy and individual adherence to MD standards. The application of the index to a Portuguese adult sample showed that most participants exhibited moderate to high adherence to MD principles, reflecting the continued presence of Mediterranean dietary habits while also identifying areas for improvement, particularly regarding the inclusion of ultra-processed and non-local ingredients.

Overall, the MedRI is a pioneering, evidence-based tool that links nutrition science, gastronomy, and sustainability assessment. It offers valuable applications for researchers, educators, health professionals, and policymakers, supporting the promotion of healthier and more sustainable dietary habits. Future work should focus on expanding its validation across diverse cultural and geographic settings, exploring digital integration for dietary tracking and education, and implementing the MedRI in nutrition education and public health programs aimed at preserving and enhancing Mediterranean food heritage.

## Figures and Tables

**Table 1:** Mediterranean Diet Recipe Index (MedRI).

			Recipe Category						
Component	Criteria	Points	Breakfast	Soup	Main Dish	Vegetarian Dish	Vegan Dish	Salad, Sauces, and Side Dish	Dessert
**I. Recipe composition dimension**									
Soup, stews, and casseroles	Yes	2 pts		x	x	x	x	x	
Sofrito (garlic, onion, tomato, and olive oil)	Yes	2 pts			x	x	x	x	
Vegetables	Yes	2 pts		x	x	x	x	x	x
Fresh fruit (including tomato)	Yes	2 pts	x	x	x	x	x	x	x
Legumes	Yes	2 pts		x	x	x	x	x	x
Whole grains and unrefined cereals	Yes	2 pts	x		x	x	x	x	x
Nuts	Yes	2 pts	x		x	x	x	x	x
Olive oil	Yes	2 pts		x	x	x	x	x	x
Aromatic herbs	Yes	2 pts		x	x	x	x	x	x
Eggs	Yes	2 pts	x		x	x		x	x
Butter and margarine	Yes	1 pts	x		x	x		x	x
Low-fat yogurt and milk	Yes	1 pts	x		x	x		x	x
Low-fat cheese	Yes	2 pts	x		x	x		x	x
Fish and seafood	Yes	2 pts			x			x	
Red meat	Yes	1 pts			x			x	
Processed meat	Yes	−1 pts	x		x			x	x
White meat	Yes	2 pts			x			x	
Sugar	Yes	−1 pts	x		x	x	x	x	x
**II. Consumption context dimension**									
Was this recipe made to share with others?	Yes	2 pts	x	x	x	x	x	x	x
Of the ingredients used, what were the selection criteria?									
Purchased from local markets, own production, directly from the producer, national products	Yes	2 pts	x	x	x	x	x	x	x
Non-national products	Yes	−1 pts	x	x	x	x	x	x	x
Seasonal products	Yes	2 pts	x	x	x	x	x	x	x
Ultra-processed products	Yes	−1 pts	x	x	x	x	x	x	x
Fresh products	Yes	2 pts	x	x	x	x	x	x	x
Certified products (Organic, PDO …)	Yes	2 pts	x	x	x	x	x	x	x

“x” indicates the presence of the corresponding component in the recipe.

**Table 2:** Individual adherence score to MD.

	Index Classification		
Recipe Category	Red	Yellow	Green
Breakfast	−4 to 0	1 to 3	4 to 20
Soup	−2 to 2	3 to 5	6 to 22
Main dish	−4 to 4	5 to 10	11 to 39
Vegetarian dish	−3 to 4	5 to 12	13 to 34
Vegan dish	−3 to 4	5 to 10	11 to 28
Salad, sauces, and side dish	−4 to 3	4 to 8	9 to 39
Dessert	−4 to 2	3 to 7	8 to 30

**Table 3:** Recipes’ adequacy score to MD.

	Index Classification		
Recipe Category	Red	Yellow	Green
Breakfast	−2 to 0	1 to 3	4 to 10
Soup	0 to 2	3 to 5	6 to 12
Main dish	−2 to 4	5 to 10	11 to 29
Vegetarian dish	−1 to 4	5 to 12	13 to 24
Vegan dish	−1 to 4	5 to 10	11 to 18
Salad, sauces, and side dish	−2 to 3	4 to 8	9 to 29
Dessert	−2 to 2	3 to 7	8 to 20

**Table 4:** Minimum, maximum, mean value, and standard deviation for quantitative variables.

Variable	Minimum	Maximum	Mean	Std. Deviation
Age (years)	18	74	44.99	±13.56
Height (cm)	147.00	188.00	165.79	±7.97
Weight (kg)	38.00	168.00	69.23	±14.90
BMI (kg/m^2^)	15.61	59.52	25.11	±4.82

**Table 5:** Sociodemographic characterization.

Variable		N	%
Age class	Adults (18–30 years)	40	16.5
	Middle-aged adults (31–50 years)	106	43.4
	Senior adults (51–65 years)	81	33.2
	Older adults (aged 66 years or more)	15	6.1
	No answer	2	0.8
Gender	Female	193	79.1
	Male	51	20.9
Education level	Basic school (9th year)	5	2.0
	Secondary school (12th year)	34	13.9
	University graduation	117	48.0
	Post-graduate studies (Master’s/PhD)	86	35.2
	Other	1	0.4
	No answer	1	0.4
Living environment	Rural	53	21.7
	Urban	132	54.1
	Suburban	57	23.4
	No answer	2	0.8
BMI Class	Underweight (<18.5 Kg/m^2^)	6	2.5
	Normal weight (18.5–24.9 Kg/m^2^)	130	53.3
	Overweight (25.0–29.9 Kg/m^2^)	69	28.3
	Obesity (>30 Kg/m^2^)	31	12.7
	No answer	8	3.3
Exercise	Never	16	6.6
	Sporadically (Less than 1/week)	41	16.8
	Occasionally (1/week)	54	22.1
	Moderately (2–3/week)	91	37.3
	Intensively (+3/week)	41	16.8
	No answer	1	0.4
Total		244	100%

**Table 6:** Chi-square, contingency coefficient, Cramer’s V and Cohen’s kappa.

Recipe Category	Chi-Square Sig.	Contingency Coefficient	Cramer’s V	Cohen’s Kappa
Breakfast	*	*	*	*
Soup	*	*	*	*
Main dish	<0.001	0.640	0.590	0.059
Vegetarian dish	0.059	0.511	0.595	0.006
Vegan dish	0.248	0.500	0.577	0.200
Salad, sauces, and side dish	0.782	0.071	0.071	0.010
Dessert	0.028	0.612	0.775	0.273

* For these recipe categories there is no distribution of individual adherence score to an MD, since all cases fall into the green category; thus, it is not possible to compute the statistics.

**Table 7:** MedRI individual adherence score.

	Index Classification			
Recipe Category	Red	Yellow	Green	N
Breakfast	−	−	100%	8
Soup	−	−	100%	17
Main dish	8.6%	23.9%	67.5%	163
Vegetarian dish	−	43.8%	56.3%	16
Vegan dish	−	50.0%	50.0%	4
Salad, sauces, and side dish	−	6.7%	93.3%	15
Dessert	−	62.5%	37.5%	8

**Table 8:** MedRI recipes’ adequacy score.

	Index Classification			
Recipe Category	Red	Yellow	Green	N
Breakfast	−	62.5%	37.5%	8
Soup	−	23.5%	76.5%	17
Main dish	23.2%	64.0%	12.8%	164
Vegetarian dish	18.8%	68.8%	12.5%	16
Vegan dish	25.0%	75%	−	4
Salad, sauces, and side dish	−	93.3%	6.7%	15
Dessert	50.0%	50.0%	−	8

**Table 9:** Correlation between sociodemographic characteristics and MedRI scores.

Variable	Correlation with MedRI Individual Adherence Score to an MD	Correlation with MedRI Recipes’ Adequacy Score to an MD
Age class	0.130 *	0.112
Gender	−0.162 *	−0.150 *
Education level	0.239 **	0.221 **
Living environment	−0.005	−0.039
District	−0.044	−0.053
BMI Class	0.073	0.115
Exercise	−0.056	−0.062

** Significant correlation at the 0.01 level of significance. * Significant correlation at the 0.05 level of significance.

**Table 10:** Correlation between the consumption context dimension and MedRI scores.

			Ingredient Selection Criteria							
		Share Meal	Local	Non- National	Seasonal	Ultra- Processed	Fresh	Certified	MedRI Individual Adherence Score	MedRI Recipes’ Adequacy Score
	Share meal	1.000								
Ingredient selection criteria	Local	0.227 **	1.000							
	Non-national	−0.056	−0.012	1.000						
	Seasonal	0.105	0.096	0.104	1.000					
	Ultra-processed	0.098	0.067	0.085	0.073	1.000				
	Fresh	0.046	0.123	0.015	0.326 **	−0.037	1.000			
	Certified	−0.135 *	0.110	0.008	0.136 *	0.050	0.119	1.000		
	MedRI individual adherence score	0.209 **	0.274 **	0.100	0.475 **	0.076	0.478 **	0.151 *	1.000	
	MedRI recipes’ adequacy score	0.092	0.078	0.031	0.161 *	0.029	0.194 **	−0.081	0.869 **	1.000

** Significant correlation at the 0.01 level of significance. * Significant correlation at the 0.05 level of significance.

**Table 11:** Factor analysis of ingredient selection criteria.

Factor	VE ^1^	Ingredient Selection Criteria	Loading
F1	15.16%	National products	0.625
		Seasonal products	0.544
		Fresh products	0.811
F2	10.44%	Protected Designation of Origin	0.690
		Protected Geographical Indication	0.841
F3	10.03%	Organic products	0.709
		Buying directly from the producer	0.610
		Ultra-processed foods	−0.489
F4	8.94%	Foreign products	0.712
		Own production	−0.687
F5	8.49%	Economic products	0.793
		Non-certified products	0.581
F6	8.14%	Buying in local markets	0.845

^1^ VE = Variance explained.

## Data Availability

The original contributions presented in the study are included in the article, and further inquiries can be directed to the corresponding author.
